# Insecticide-treated nets ownership and utilization among under-five children following the 2010 mass distribution in Burkina Faso

**DOI:** 10.1186/1475-2875-13-353

**Published:** 2014-09-04

**Authors:** Souleymane Diabaté, Thomas Druetz, Emmanuel Bonnet, Seni Kouanda, Valéry Ridde, Slim Haddad

**Affiliations:** University of Montreal Hospital Research Centre (CRCHUM), Montreal, Canada; Department of Social and Preventive Medicine, University of Montreal, Montreal, Canada; Institut de Recherche pour le Développement (IRD), Ouagadougou, Burkina Faso; Institut de Recherche en Sciences de la Santé (IRSS) du CNRST, Ouagadougou, Burkina Faso

**Keywords:** Malaria, Under-five children, Saturation with bed nets, Vector control, Burkina Faso

## Abstract

**Background:**

Periodic mass distributions contribute significantly to universal access to insecticide-treated nets (ITNs). However, due to the limited number of nets distributed, needs remain unsatisfied, particularly in large households.

**Methods:**

This study was conducted in Kaya health district following the 2010 mass distribution of ITNs in Burkina Faso. Data were collected on the socio-economic and geo-spatial characteristics and ITN possession and utilization levels of 2,004 households. The study explored: 1) ITN access, in terms of intra-household saturation with ITNs (households with at least one ITN for every two members) correctly installed and in very good physical condition; and 2) factors influencing the decision to place under-five children under a net. Particular attention was given to vector control activities undertaken by mothers.

**Results:**

Of the 2,004 households, 90% possessed at least one ITN. However, intra-household saturation with ITNs was below 60% in small households and below 20% in large ones (>6 members). Crude proportion ratios comparing possession and levels of intra-household saturation with ITNs varied between 1.5 (small households) and 7.8 (large households). The proportions of households with ITNs for every two members that were correctly hung or in very good physical condition ranged from 0% to 6.5% in large households and 27.8% to 40.7% in small ones. ITN use to protect under-five children was lower in large households; it was significantly higher when there was at least one ITN for every two members. In large households, it was significantly higher when a child had experienced an episode of any illness in the previous two weeks and when the mother had taken actions to control vector proliferation. In small households, ITN use was significantly higher in families with agricultural land and children aged 12–23 months.

**Conclusion:**

Ownership rates were high, but real access to bed nets remained limited. The allocation process disadvantages large families. Real access to bed nets implies they are available, properly installed, and in good condition. More post-campaign awareness-raising activities targeting preventive practices in households could foster more effective ITN use.

## Background

Malaria remains one of the leading causes of morbidity and mortality worldwide [1, 2]. Sub-Saharan Africa is the most affected region, with more than 78% of all deaths occurring among under-five children [1]. In Burkina Faso, malaria is responsible for 22% of all deaths reported in medical centres, and 60% of those malaria-related deaths occur among under-five children [3, 4]. Insecticide-treated nets (ITNs) and indoor residual spraying (IRS), along with artemisinin-based combination therapy (ACT), are the cornerstones of vector control and malaria elimination [1, 5, 6]. Studies have reported a sizeable decline in malaria prevalence and mortality in sub-Saharan Africa following deployment of ACT and distribution of ITNs [5, 7, 8]. Between 2001 and 2010, malaria control interventions have helped prevent approximately 842,800 deaths among African children [9].

To accelerate universal access to bed nets, routine distribution of ITNs is often reinforced by periodic mass donations of fixed numbers of nets. Campaigns are aimed at increasing coverage—the proportion of households owning ITNs—and reducing social inequalities in bed net ownership and use [10–13]. However, due to the insufficient number of nets distributed, the needs of households, particularly large ones, are often not fulfilled. The possession of at least one net for every two members of the household is seldom achieved in large households [12, 14]. For example, the proportion of households owning at least one ITN was 91% after the 2011 universal coverage campaign in Tanzania. At the same time, only 35% of households had at least one ITN for every two members [14].

In Burkina Faso, eight million ITNs were distributed between July 2009 and January 2010 [4, 15]. Before the distribution, the number of members per household was calculated through a pre-campaign survey, and the plan was to give households one net for every two members. Unfortunately, the total number of ITNs acquired for the campaign was lower than projected. Accordingly, the national authorities decided, once the distribution was launched, to subtract one ITN for households with more than four members [4, 15].

Six months after the distribution campaign, this study was conducted in Kaya, in north-central Burkina Faso, to examine levels of accessibility to ITNs and their use among under-five children. Rather than focusing only on households’ possession of ITNs, the analysis of accessibility examined the extent to which household members’ needs were satisfied, as measured by an indicator reflecting intra-household saturation coverage in ITNs: the proportion of households owning at least one ITN for every two persons [14, 16], the proportion of correctly installed ITNs, and the proportion of ITNs in very good condition. These analyses examined both small and large households. The analysis of ITN use compared utilization levels according to geographic context. The main factors influencing the decision to place an under-five child under an ITN were also studied. To explore prevention practices, particular attention was given to the link between placing a child under an ITN and mothers’ actions to curb vector proliferation.

## Methods

### Study site

Six months after the end of the campaign, a cross-sectional survey was conducted during the highest malaria transmission period of the rainy season (August–September 2011). The survey took place in the area covered by a population observatory—the Kaya Health and Demographic Surveillance System (Kaya HDSS) [17]. For the purpose of the study, a subsample of the panel of households surveyed by the observatory was used. The sample included 2,004 households randomly selected and stratified between urban sectors and rural areas (18 villages located within a radius of 20 kilometres around the city).

### Data collection and definition of main variables

Information on the households’ socio-economic, geographic, and spatial characteristics (socio-economic indicators, longitude and latitude, roads, lakes and sources of stagnant water) was extracted from the Kaya HDSS database. Households were geo-referenced through a global positioning system, and the villages’ geographic boundaries were delimited by means of households’ geographic centres and Thiessen polygons [18]. The ITNs owned by each household were numbered and, when allowed by the head of the household, the surveyor observed whether they were correctly hung (placement) and in very good physical condition. Surveyors were specifically trained for these observations. An ITN is correctly installed over the sleeping place when the four hanging points are used, it is at an appropriate height, and it fully covers the sleeping place, with no space between the net and the edges of the sleeping place. An ITN in good physical condition is one with no tears and no stretching of the standard mesh. Mothers were asked whether each child had slept under an ITN on the night preceding the survey. They also answered questions related to their knowledge, attitudes, and practices related to malaria prevention and treatment. Three of these questions concerned actions undertaken regularly to control vector proliferation: cleaning the house, eliminating stagnant water, and eliminating larval sites around the house. All under-five children were examined and their episodes of any illness over a two-week recall period were recorded.

Eight indicators of access and use for each household or eligible child were assessed: *ITN ownership*: 1) proportion of households with at least one ITN (the denominator being the total number of households surveyed); 2) intra-household saturation with ITNs, measured by the proportion of households with at least one ITN for every two people (the numerator being the number of households with at least one ITN for every two people and the denominator being the total number of households owning at least one ITN) [16]. *ITN placement status* above the sleeping place: the proportion of households with 3) all ITNs correctly hung; 4) at least one ITN correctly hung; 5) at least one ITN for every two people correctly hung. *ITN physical condition:* the proportion of households with 6) all ITNs in very good condition; 7) at least one ITN in very good condition; and 8) at least one ITN in very good condition for every two people; *ITN use to protect under-five children:* 8) number of under-five children who had slept under a bed net the previous night.

### Data analysis

Analyses of ITN access were stratified according to urban/peri-urban/rural areas [19] and household size (six members or less, and more than six). Crude proportion ratios (CPRs) were computed to compare: 1) household ITN ownership and intra-household saturation with ITNs (the proportion of households with at least one ITN divided by the proportion of households with at least one ITN for every two people); and 2) proportion of households with at least one ITN hung/in very good physical condition and proportion of households with at least one ITN for every two members hung/in very good physical condition (the denominator being the proportion of households with at least one ITN for every two members hung/in very good physical condition). Utilization rates among under-five children were also stratified by urban/peri-urban/rural areas and household size. Factors associated with use were studied through multilevel regression models, with children being nested within households and villages or urban sectors. Analyses were conducted using Arcgis 10.x ESRI [20] and Stata 13 software [21].

The study was approved by the Ethics Committee of the University of Montreal Hospital Research Centre and the Health Research Ethics Committee in Burkina Faso.

## Results

### Sample and ITN characteristics

The survey covered 2,004 households, 1,906 children, and 4,811 ITNs (Table 1). To reduce vector proliferation, mothers in half of the households had undertaken at least one of these actions: cleaning the house and/or eliminating stagnant water and/or eliminating larval sites around the house. There were no noticeable differences between urban, rural, and peri-urban settings. Nearly one child out of three had presented an episode of any illness during the two-week recall period. The great majority (90%) of ITNs found in the homes were acquired during the campaign.

**Table 1:** **Sample characteristics**

Characteristic (proportion; % or number; n)	Overall	Small household			Large household		
		(≤6 people)			(>6 people)		
		Rural	Peri-urban	Urban	Rural	Peri-urban	Urban
**Households (n)**	2004	402	143	566	414	104	373
***General characteristics***							
Median number of members (interquartile range)	6(4-9)	**5 (4-6)**	**4 (3-5)**	**4 (3-5)**	**9 (8-12)**	**9 (8-11)**	**9 (7-11)**
Own agricultural land	52.2	**67.4**	**49.0**	**21.9**	**85.8**	**74.0**	**40.0**
Own any cattle^†^	74.0	**88.1**	**77.6**	**41.7**	**97.1**	**90.4**	**76.1**
Access to private toilets	61.0	**26.6**	**41.3**	**95.1**	**29.0**	**43.3**	**94.4**
Access to safe drinking water	49.7	**14.4**	**29.4**	**86.6**	**14.0**	**31.7**	**84.2**
Distance to the nearest lake or stagnant water (<1000 versus ≥1000 metres)	40.1	**35.8**	**28.7**	**45.6**	39.1	32.7	44.0
***Vector control actions undertaken by the mother***							
House cleaning	40.6	40.3	41.3	40.6	41.3	45.2	38.3
Elimination of stagnant water	20.3	18.9	21.0	20.9	20.5	19.2	20.6
Elimination of larval sites	28.3	28.9	24.5	28.8	27.5	28.9	29.2
Indicator = at least one of these three actions	50.4	50.5	49.7	50.4	49.5	58.7	49.3
**Under-five children (n)**	1,906	313	102	355	576	156	404
Proportion of under-five among all children present during the survey	79.7	82.2	82.9	83.3	**77.4**	**85.3**	**75.8**
At least 1 episode of any illness during the preceding 2 weeks^‡^	30.6	30.0	29.7	31.9	29.2	25.0	35.0
**Insecticide-treated nets (n)**	4,811	766	262	922	1,430	311	1,119
Acquired from the campaign	93.7	**96.7**	**96.6**	**86.6**	**98.0**	**93.3**	**91.7**
Age ≤12 months	96.6	**97.9**	**97.3**	**92.7**	**98.7**	**97.4**	**95.8**
ITNs observed	62.5	66.6	69.5	70.4	56.4	57.9	60.7

Notes: ITNs = insecticide-treated nets; Proportions or numbers in bold = P value <0.05 (comparing urban/peri-urban/rural areas); ^**†**^Beef, camel, goat, horse, pig or sheep; ^‡^Among those present during the survey (n = 1,721).

### Access to ITNs

**Figure 1:**
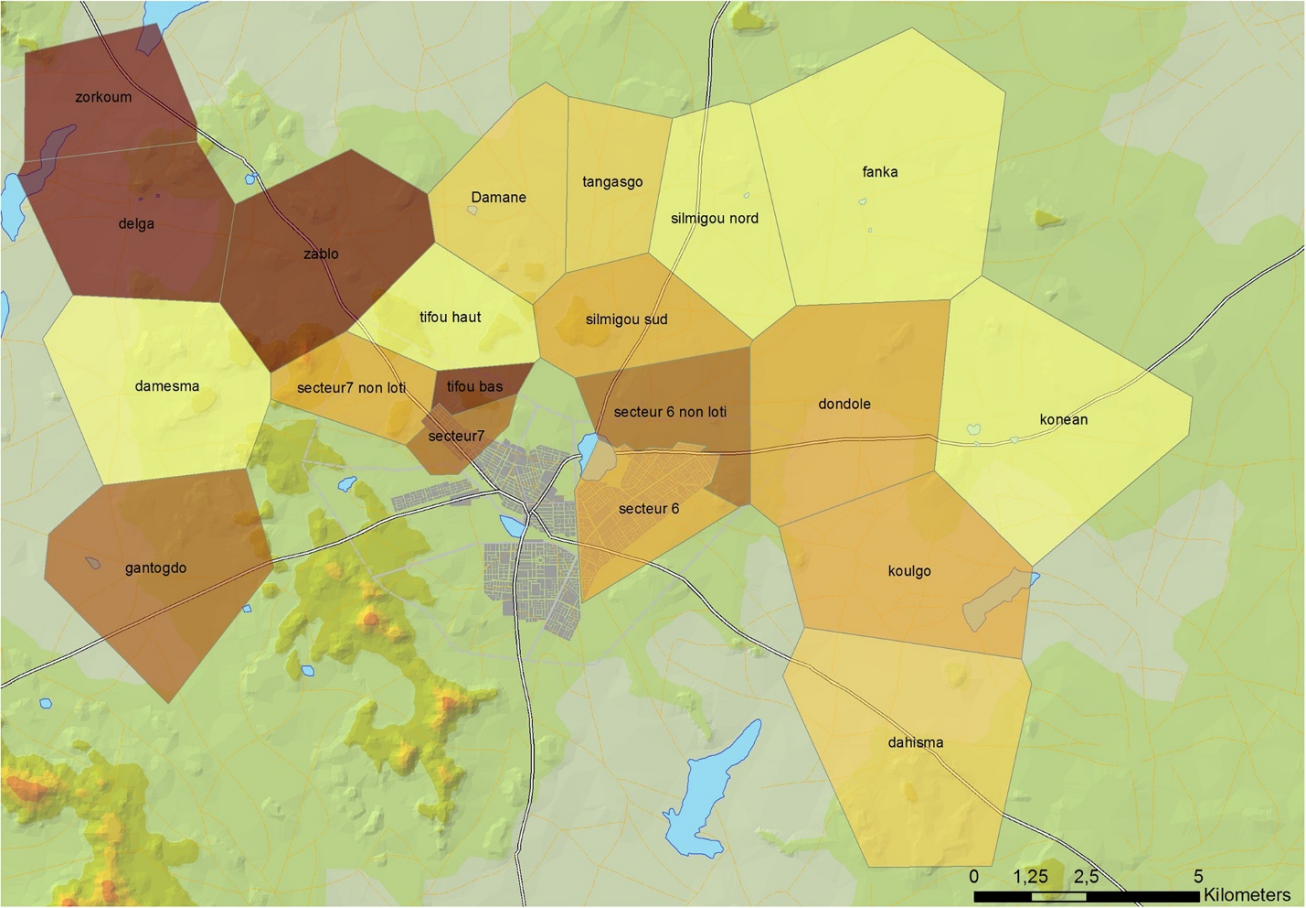
**Intra-household saturation with ITNs by village/sector: proportion of households owning at least one insecticide-treated net for every two members.** This ownership indicator is not uniformly distributed (p value <0.05). There are disparities across villages/sectors.

Ninety percent of all households possessed at least one ITN. Ownership increased with household size and in rural areas, where transmission is higher (Table 2). There were more than two members per ITN in less than one-third of small households, but in 80% of large ones. Consequently, the gap between possession and intra-household saturation with ITNs was substantial, especially in large households. All crude proportion ratios were highly significant, varying between 1.5 in peri-urban small households and 7.8 in rural large households. Overall, intra-household saturation with ITNs was heterogeneous across villages (Figure 1) and urban sectors (p = 0.046).

**Table 2:** **Access to and characteristics of the insecticide-treated nets (ITNs)**

ITN indicator (proportion, %; otherwise indicated)	Overall	Small household (≤6 people)			Large household (>6 people)		
		Rural	Peri-urban	Urban	Rural	Peri-urban	Urban
**Ownership** ^†^							
Median number (IQR)	2 (2-3)	**2 (2-2)**	**2 (1-2)**	**2 (1-2)**	**3 (3-4)**	**3 (2-4)**	**3 (2-4)**
Access: at least 1 ITN (a)	89.9	**93.8**	**90.9**	**83.6**	**96.1**	**91.4**	**87.7**
Intra-household saturation with ITNs: at least 1 ITN for every 2 people (b)	37.0	56.2	60.8	54.1	12.3	12.6	18.0
*CPR (95% CI) = a/b*	*2.4 (2.3-2.6)*	*1.7 (1.5-1.8)*	*1.5 (1.3-1.7)*	*1.5 (1.4-1.7)*	*7.8 (6.0-10.2)*	*7.2 (4.2-12.3)*	*4.9 (3.9-6.2)*
**Placement status** ^‡^							
All ITNs correctly hung	46.5	50.4	56.4	54.6	38.5	43.2	36.3
At least 1 ITN correctly hung (c)	78.6	77.4	80.2	80.3	79.1	83.8	74.6
At least 1 ITN for every 2 people correctly hung (d)	18.1	27.8	33.7	30.9	4.1	0	3.2
*CPR (95% CI) = c/d*	*4.3 (3.9-4.9)*	*2.8 (2.3-3.4)*	*2.4 (1.8-3.2)*	*2.6 (2.2-3.1)*	*19.2 (11.0-33.5)*	*-*	*23.1 (11.7-45.9)*
**Physical integrity** ^‡^							
All ITNs in very good condition	62.1	68.1	67.3	71.6	53.3	44.6	54.8
At least 1 ITN in very good condition (e)	92.0	**93.8**	**85.2**	**92.4**	92.4	86.5	93.2
At least 1 ITN for every 2 people in very good condition (f)	25.1	40.3	39.6	40.7	6.5	4.1	6.5
*CPR (95% CI) = e/f*	*3.7 (3.3-4.0)*	*2.3 (2.0-2.7)*	*2.2 (1.7-2.8)*	*2.3 (2.0-2.6)*	*14.2 (9.2-21.9)*	*21.3 (7.0-64.9)*	*14.4 (9.0-23.2)*

Notes: CPR = crude proportion ratio; 95% CI, = 95% confidence interval; a, denominator = all households; b, among households owning at least one ITN; Proportions or numbers in bold = p value < 0.05 (comparing urban/peri-urban/rural areas); ^†^Assessed taking into account all the 4,811 ITNs reported; ^‡^Assessed taking into account only the 3,006 ITNs observed by the surveyors.

Surveyors were allowed by household heads to observe about two-thirds of the ITNs (Table 1). Half of the ITNs seen were correctly hung (Table 2), while at least one ITN was correctly hung in about four out of five households. The proportion of ITNs correctly hung for every two persons was much lower, ranging from zero in large households in peri-urban areas to about one-third in small households in those areas. Among small households, the proportion with one ITN correctly hung was two times higher than the proportion of households with at least one ITN for every two members correctly hung. The crude proportion ratio reached 19 among large households.

In two-thirds of the homes visited, all the ITNs observed were in very good physical condition (Table 2). But here, too, values decreased substantially when the indicators took into consideration saturation. The ratio between the proportion of households having at least one ITN in very good condition and the proportion of households with one ITN for every two people in very good physical condition and the proportion of households having at least one ITN in very good condition was two in small households and 14 to 20 in large ones. The proportion of households with impaired ITNs was not significantly different between rural, peri-urban, and urban areas.

### Use of ITNs to protect under-five children

**Figure 2:**
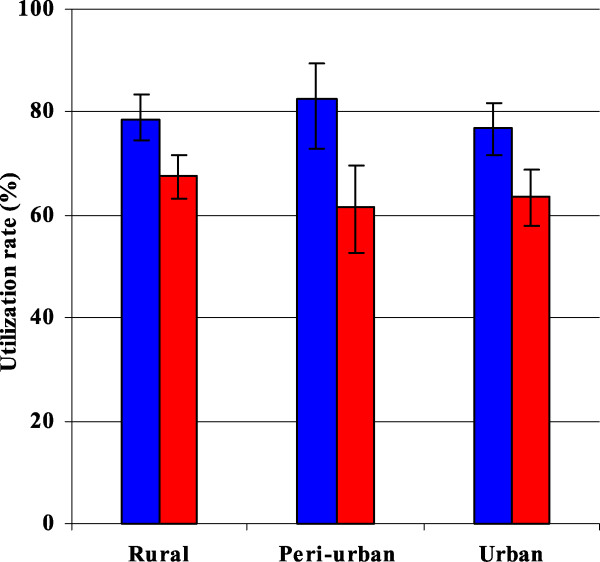
**Proportion of under-five children placed under Insecticide-treated nets the night before the survey.** Blue, small household (≤6 members); Red, large household (>6 members); In each residential area, the differences in utilization levels by household size are statistically significant (all p values <0.05).

Among households possessing at least one ITN, 70% of the under-five children had been placed under an ITN the night preceding the survey. There was no significant difference in level of use by location of residence (rural, peri-urban, urban) or household size (small, large). However, in each of the three settings, the use of ITNs was significantly lower in large households than it was in small ones (Figure 2). Statistical models conducted on the subsample of large households showed that use of ITNs was significantly higher when there was at least one ITN for every two members, when the child had an episode of illness in the preceding two weeks, and when the mother had taken actions to control vector proliferation (Table 3). Use in small households was significantly higher when the family held agricultural land (a proxy for living in an environment that favours vector proliferation) and when the child was 12 to 23 months of age. The proportion of children who slept under an ITN was not significantly associated in this survey with variables such as residential area, cattle ownership, access to safe drinking water or private toilets, and proximity to lakes or stagnant water.

**Table 3:** **Insecticide-treated nets (ITN) utilisation to protect under-five children**

	Model 1:	Model 2:
	Small household	Large household
	(≤6 members, n = 647)	(>6 members, n = 959)
**Fixed effects**		
**Intercept (estimate, SE)**	0.55 (0.51)	0.22 (0.46)
**OR (95%CI)**		
**Intra-household saturation with ITNs: At least 1 ITN for every 2 people** ^**†**^	**1.6 (1.02**–**2.58)**	**1.9 (1.07**–**3.42)**
**Vector control activities** ^‡^	0.9 (0.58–1.44)	**1.5 (1.07**–**2.16)**
**At least 1 episode of child illness during the preceding 2 weeks** ^**₱**^	1.5 (0.92–2.56)	**1.5 (1.03**–**2.12)**
**Residential area**		
Peri-urban versus urban	1.4 (0.59–3.33)	0.9 (0.47–1.69)
Rural versus urban	1.0 (0.41–2.22)	1.2 (0.67–2.14)
**Ownership of agricultural land**	**1.9 (1.10**–**3.30)**	0.9 (0.57–1.32)
**Ownership of any cattle** ^**₤**^	0.8 (0.48–1.41)	1.3 (0.70–2.24)
**Access to safe drinking water**	1.2 (0.60–2.30)	1.2 (0.75–1.94)
**Access to private toilets**	0.9 (0.45–1.62)	0.8 (0.49–1.15)
**Distance to nearest lake or stagnant water point (<1000 vs. ≥1000 m)**	1.3 (0.80–2.17)	0.9 (0.65–1.32)
**Children’s age** (months; reference = 0–11)		
12–23	**2.2 (1.10**–**4.55)**	1.3 (0.78–2.28)
24–35	2.0 (0.98–3.94)^*^	1.1 (0.66–1.82)
36–47	1.6 (0.81–3.11)	1.1 (0.67–1.84)
48–59	1.3 (0.80–2.17)	1.0 (0.60–1.75)
**Random effect**		
Household (estimate, SE)	0.91 (0.40)	0.96 (0.20)
Intra-class correlation (95% CI)	20.1 (4.3–58.7)	21.7 (10.8–39.0)

Notes: SE = Standard error; OR = Odds ratio; 95% CI = 95% confidence interval; Bold = p value <0.05; ^*^P value <0.10; ^**†**^Among households owning at least one ITN, unadjusted OR (95% CI) = 1.71 (1.09–2.69) in small households and 1.84 (1.04–3.27) in large ones; ^‡^Cleaning house, elimination of stagnant water and larval sites around the house by the mother of the under-five children; ^₱^Any illness; ^₤^Beef, camel, goat, horse, pig or sheep.

## Discussion

The majority of the households (90%) owned bed nets. However, there was a large gap between ownership of at least one ITN and intra-household saturation with ITNs. As anticipated, the needs in bed nets remained clearly unfulfilled, particularly in large households, where there was very low intra-household saturation with ITNs (20%). With this low coverage, limited impacts on malaria transmission are to be expected, even if bed net use were optimal. Low saturation rates in large households following the distribution of a fixed number of bed nets have also been found in Tanzania [14]. The allocation process needs to be rethought and based on household size to increase access to bed nets in crowded households [22]. ITN placement and physical condition were better in small households, where intra-household saturation with ITNs was higher and competition for nets less perceptible. Therefore, providing more nets per household would likely enhance their placement [23] and preserve their physical condition against rapid deterioration.

These results offer an opportunity to reflect on how to estimate ITN needs and monitor the outcomes of mass campaigns. Intra-household saturation with ITNs, even though it is an imperfect indicator, is clearly a more informative indicator of access than the commonly used measure of possession of at least one ITN per household [8, 13] which does not take into account household size or sleeping places and consequently does not precisely indicate the extent to which individuals have real access to the nets [11, 12, 14]. The gap between ownership rates per household and intra-household saturation with ITNs is so large that monitoring access to bed nets based on the former could be misleading. Similarly, the proportion of households with at least one ITN hanging or in very good physical condition also overestimates individuals’ access to bed nets and, ultimately, population levels of protection. Here, too, saturation-based indicators might be more accurate in monitoring access to bed nets. Finally, because ITN access depends on fulfillment of needs, proper placement, and good physical condition of the nets, and not just on the level of household ownership, an accurate understanding of access to bed nets and the assessment of campaign outcomes should be based on appropriate measurements of these three components.

Depending on the geo-spatial context and the size of the household, two-thirds to three-quarters of under-five children had slept under an ITN the previous night. As has been observed in other studies, more children were placed under ITNs in households with higher saturation rates [12, 24, 25]. In other words, children’s protection is clearly linked to access to bed nets. ITN use was also higher in households where mothers cleaned the house and eliminated stagnant water and larval sites in the homestead. This suggests that actions by mothers to decrease mosquito density tend to be undertaken jointly with actions to protect the child and might reflect a more general concern among mothers about malaria prevention at home. Further research will eventually help in better understanding the web of relationships between vector control actions, ITN use, and malaria morbidity.

Children who had recent episodes of any illness were more likely to have been placed under bed nets the night before the survey. The reasons behind that observation are not yet clear and would require further investigations. It may be that some parents placed children presenting an episode of fever under the nets because they believed it would prevent progression of the episode to severe states [26–28]. Further qualitative studies are needed to understand the relationship between bed net use and children’s conditions.

ITNs were used more for children aged 12–23 months than for those under 12 months, particularly in small households. Sleeping arrangements can partly explain this result. Baume and Marin reported that parents consider children under 24 months of age to be more vulnerable to malaria and often place them in priority under bed nets [24]. Finally, ITN use was not associated with residential area or household characteristics, such as cattle ownership or access to safe drinking water or private toilets. This result was expected because, apart from not taking into account household size, mass distribution campaigns of fixed numbers of bed nets generally reduce inequities in ownership and use [10, 13].

### Limitations

Because there were no data available before the campaign, the extent to which the 2010 distribution campaign improved ITN coverage and use in the communities surveyed was not estimated. Such a baseline would have been helpful in assessing changes in ITN access and use attributable to the campaign. A second limitation was in the set of covariates available for the study of factors associated with the ITN use. In particular, it is common to check for the influence of mother’s education on use. Unfortunately, there were too many missing values on education in the dataset to incorporate this variable into the model. However, the rate of education of adult women in these communities is low (only 12.5% of mothers had gone to school), and it is expected that education would not be a strong modifier in the model. Finally, there are reasons to believe the rates of ITNs hung and of ITNs in very good condition were somewhat overestimated. Often, surveyors were not allowed to enter the home and observe the bed nets themselves (37.5% of the ITNs were not observed). It is reasonable to assume that in some cases, respondents did not want to let the surveyor see bed nets that were not hung or that were impaired.

## Conclusion

From this study, some lessons related to the recent mass distribution campaign can be derived. First, ownership rates were high, but real access to bed nets remained limited. The allocation process clearly acted as a constraint that disadvantaged large families, who tend to live in rural, highly exposed areas. The allocation process should achieve acceptable levels of intra-household saturation with ITNs and fulfill populations’ needs in terms of protection against malaria transmission. Second, real access to bed nets implies not only that they are available, but also that they are properly installed and in good condition. In general, campaign organizers do not pay enough attention to these critical issues. Finally, more post-campaign sensitization initiatives targeting mothers’ awareness and preventive practices could eventually contribute to more effective use of bed nets and higher levels of protection against transmission.
